# Natural Products for the Treatment of Obesity, Metabolic Syndrome, and Type 2 Diabetes

**DOI:** 10.1155/2013/871018

**Published:** 2013-12-04

**Authors:** Menaka C. Thounaojam, Srinivas Nammi, Ravirajsinh Jadeja

**Affiliations:** ^1^National Brain Research Centre, Manesar, Gurgaon 122 051, India; ^2^School of Science and Health, University of Western Sydney, NSW 2751, Australia; ^3^Centre for Complementary Medicine Research, University of Western Sydney, NSW 2751, Australia; ^4^Division of Gastroenterology/Hepatology, Department of Medicine, Medical College of Georgia, Georgia Regents University, Augusta, GA 30912, USA

Globally, the incidence of obesity, metabolic syndrome, and diabetes (OMD) are continuously on the rise because of rapid changes in human life-style and dietary habits. Herbal extracts are of special interest in treating combination of these diseases because of their multipronged mode of action. The list of potential herbals to control metabolic diseases is ever-expanding. However, because of poor characterization and safety issues, these herbs face limitations for their clinical usage. This special issue is a collection of research and review articles on preclinical and clinical benefits of herbals in controlling OMD. This special issue contains 24 articles accepted from a total of 37 submissions consisting of 20 research articles, 3 review articles, and 1 clinical study. The research articles in this issue can be broadly divided into three disease categories—nonalcoholic steatohepatitis (NASH), obesity, and diabetic complications.

Four articles of this special issue focus on evaluating the protective role of herbal extracts on NASH. The studies by X. R. Yang et al. “*Effect of dietary cocoa tea (Camellia ptilophylla) supplementation on high-fat diet-induced obesity, hepatic Steatosis, and hyperlipidemia in mice*” and H.-Y. Jung et al. “*The Korean mistletoe (Viscum album coloratum) extract has an antiobesity effect and protects against hepatic steatosis in mice with high-fat diet-induced obesity*” report the potential benefits of *Camellia ptilophylla *and *Viscum album coloratum* extracts against HFD-induced NASH. Another two articles evaluated protective effects of biherbal combination (*S. miltiorrhiza*, *G. jasminoides* and Grape Pomace, Omija Fruit) in ameliorating experimental NASH.

This special issue also contains five articles that focus on antiobesity potential of herbal extracts. These detailed studies evaluated the benefits of 10 herbs and their potential mechanisms responsible in controlling obesity using experimental HFD-fed mice/rat *in vivo* and 3T3L1 preadipocyte *in vitro* models. Modulation of PPAR*γ* was a key antiobesity mechanism of *Artemisia iwayomogi*, *Codonopsis lanceolata*, *Populus balsamifera* and its active component (salicortin), and beta-glucan-rich extract from *Pleurotus sajor-caju* (Fr.) Singer. H.-Y. Shin et al. reported an activation of AMP-activated protein kinase by extract of six herbal medicines (OB-1).

In this special issue, 11 articles focus on the potential benefits of various herbal extracts/phytocompounds on diabetes-induced insulin resistance, nephropathy, retinopathy, cardiomyopathy, and inflammation. A. l. Al-Malki showed oat extract to be beneficial for diabetic nephropathy and retinopathy by modulating nuclear factor kappa B (NF-kB). Inhibition of aldose reductase activity by scopoletin ameliorated cataractogenesis in galactose-fed rats (J. Kim et al.). *Boehmeria nivea* extract (S. H. Kim et al.) and swertiamarin (T. P. Patel et al.) regulated experimental insulin resistance by modulating PPAR*γ*. In another report, S. Kadan et al. evaluated effect of eight antidiabetic medicinal plants extracts on GLUT 4 translocation. The benefits of fisetin and an ayurvedic herbal formulation (Kal-1) on diabetes-induced inflammation was also reported. Further, quercetin was shown to preserve *β*-cell mass and function in fructose-fed hyperinsulinemic rats via modulating pancreatic akt/foxo1 activation. Berberine ameliorated glucose- and insulin-induced cardiomyocyte hypertrophy by modulating PPAR*α*/NO. In an interesting article by S. E. Martinez et al. pharmacometrics of an antidiabetic compound, 3-methoxypterostilbene was reported. 3-Methoxypterostilbene inhibited *α*-glucosidase and *α*-amylase activity and exhibited approximately 50% bioavailability.

Three review articles were also incorporated in this special issue. A review by Y. Liu et al. (recent updates on beneficial role of berberine in controlling NASH) provides detailed account on molecular regulation of lipid metabolism and NASH by berberine. In another review by C. D. Lorenzo et al. the use of *in vitro* and clinical approaches to assess the benefits of plant food supplements is critically discussed. The potential benefits of Kampo, a Japanese traditional medicine, in treating obesity was reviewed by J.-i Yamakawa et al. based on basic and clinical evidence.

The only clinical study as a part of this special issue focuses on evaluating body weight lowering effects of herbal extract-THI (target herbal ingredient) on exercising healthy overweight humans following a two-month randomized double-blind, placebo-controlled trial. The study reports a significant reduction in body weight indicating its potential antiobesity effect.

We envisage that this special issue will attract broad interest in the fields of obesity, metabolic syndrome, and type 2 diabetes and encourage the perusal of in-depth molecular and cellular mechanistic investigations into the use of natural products, in particular the herbal therapies for metabolic disorders and their complications.

